# Chloroplast Phylogenomics Reveals the Intercontinental Biogeographic History of the Liquorice Genus (Leguminosae: *Glycyrrhiza*)

**DOI:** 10.3389/fpls.2020.00793

**Published:** 2020-06-17

**Authors:** Lei Duan, A.J. Harris, Chun Su, Zhi-Rong Zhang, Emine Arslan, Kuddisi Ertuğrul, Phan Ke Loc, Hiroaki Hayashi, Jun Wen, Hong-Feng Chen

**Affiliations:** ^1^Key Laboratory of Plant Resources Conservation and Sustainable Utilization, South China Botanical Garden, Chinese Academy of Sciences, Guangzhou, China; ^2^Department of Botany, National Museum of Natural History, MRC 166, Smithsonian Institution, Washington, DC, United States; ^3^College of Life Science, Northwest A&F University, Yangling, China; ^4^Germplasm Bank of Wild Species in Southwest China, Kunming Institution of Botany, Chinese Academy of Sciences, Kunming, China; ^5^Department of Biology, Faculty of Science, Selçuk University, Konya, Turkey; ^6^Department of Botany and HNU, Faculty of Biology, VNU Hanoi University of Science (HUS), Hanoi, Vietnam; ^7^Laboratory of Natural Products Chemistry, College of Pharmaceutical Sciences, Ritsumeikan University, Kyoto, Japan

**Keywords:** amphitropical disjunction, Asian-Australian disjunction, biogeography, *Glycyrrhiza*, *Glycyrrhizopsis*, long-distance dispersal, *Meristotropis*, Qinghai-Tibetan Plateau uplift

## Abstract

The liquorice genus, *Glycyrrhiza* L. (Leguminosae), is a medicinal herb with great economic importance and an intriguing intercontinental disjunct distribution in Eurasia, North Africa, the Americas, and Australia. *Glycyrrhiza*, along with *Glycyrrhizopsis* Boiss. and *Meristotropis* Fisch. & C.A.Mey., comprise *Glycyrrhiza* s.l. Here we reconstructed the phylogenetic relationships and biogeographic history in *Glycyrrhiza* s.l. using sequence data of whole chloroplast genomes. We found that *Glycyrrhiza* s.l. is sister to the tribe Wisterieae and is divided into four main clades. Clade I, corresponds to *Glycyrrhizopsis* and is sister to *Glycyrrhiza* sensu Meng. *Meristotropis* is embedded within *Glycyrrhiza* sensu Meng, and these two genera together form Clades II–IV. Based on biogeographic analyses and divergence time dating, *Glycyrrhiza* s.l. originated during the late Eocene and its most recent common ancestor (MRCA) was distributed in the interior of Eurasia and the circum-Mediterranean region. A vicariance event, which was possibly a response to the uplifting of the Turkish-Iranian Plateau, may have driven the divergence between *Glycyrrhiza* sensu Meng and *Glycyrrhizopsis* in the Middle Miocene. The third and fourth main uplift events of the Qinghai-Tibetan Plateau may have led to rapid evolutionary diversification within *Glycyrrhiza* sensu Meng. Subsequently, the MRCA of Clade II might have migrated to North America (*G. lepidota*) via the Bering land bridge during the early Pliocene, and reached temperate South America (*G. astragalina*) by long-distance dispersal (LDD). Within Clade III, the ancestor of *G. acanthocarpa* arrived at southern Australia through LDD after the late Pliocene, whereas all other species (the SPEY clade) migrated to the interior of Eurasia and the Mediterranean region in the early Pleistocene. The MRCA of Clade IV was restricted in the interior of Eurasia, but its descendants have become widespread in temperate regions of the Old World Northern Hemisphere during the last million years.

## Introduction

The papilionoid legume genus *Glycyrrhiza* L. is commonly known as the liquorice genus (Polhill, 1981, 1994; LPWG, 2017). *Glycyrrhiza* is perennial herb adapting to mesophytic and xerophytic habitats; its flowering phase ranges from June to August (Li and Cui, 1998), and the seeds disperse by the spiny/hairy pods sticking to animal fur (Zhou and Jin, 2016). The liquorice genus is well-known by its morphologically diverse fruits (ovoid, oblong, or linear, rarely moniliform, according to Bao and Larsen, 2010), and long, strong roots with great economic importance. Traditionally, the roots have been widely used as an important medicinal herb in the Old World countries ranging from China and Japan to Turkey, Greece and Egypt, with the efficacy of relieving cough and phlegm (Bao and Larsen, 2010; Chinese Pharmacopoeia Commission, 2015; Dastagir and Rizvi, 2016; Zhou and Jin, 2016; Sharma et al., 2018) and have been used to make a popular candy in the West since at least the sixteenth-century (Richardson, 2008). The unique constituent of *Glycyrrhiza*, glycyrrhizin, is broadly used as a natural sweetener and a pharmaceutical agent because of its anti-inflammatory and hepatoprotective properties (Hayashi and Sudo, 2009) and extracts of *Glycyrrhiza* can also be used in the production of cosmetics, food additives, and tobacco flavors (Li and Cui, 1998; Zhou and Jin, 2016).

Despite its large economic and cultural importance, generic and species delimitation in *Glycyrrhiza* and related genera has had a torturous taxonomic history with the number of species varying from 13 (Kruganova, 1955; Meng, 2005) to 36 (Grankina, 2008). As for its two satellite genera, *Glycyrrhizopsis* Boiss. and *Meristotropis* Fisch. & C.A.Mey., Meng (2005) first merged the latter into *Glycyrrhiza* but retained the generic status of the former (referred to as “*Glycyrrhiza* sensu Meng” hereafter). Later, taxonomists treated both *Meristotropis* and *Glycyrrhizopsis* within *Glycyrrhiza* (Li, 1963; Li and Lu, 2015; Çetin, 2015), which formed “*Glycyrrhiza* s.l.” Previous molecular phylogenetic studies supported that *Glycyrrhiza* s.l. was an early branching clade of the inverted repeat-lacking clade (IRLC) of Papilionoideae (Wojciechowski et al., 2000, 2004; Lavin et al., 2005; Duan et al., 2015). With regard to the infra-generic phylogeny of *Glycyrrhiza* s.l., Yamazaki et al. (1994) resolved that *G. glabra* L. and *G. uralensis* Fisch. ex DC. are sisters according to RAPD and RFLP markers. Similarly, based on the chloroplast *rbcL* marker, two groups were recovered within *Glycyrrhiza*: one group included *G. glabra*, *G. uralensis* and *G. inflata* Batalin and the other contained *G. echinata* L. and *G. pallidifolia* Maxim. (Hayashi et al., 1998, 2000; Hu and Chang, 2003). Hayashi et al. (2005) and Meng (2005) discovered that *G. lepidota* Pursh diverged first in the genus, while other studies suggested *G. flavescens* Boiss. was sister to the rest of *Glycyrrhiza* s.l. (Erayman et al., 2014; Çetin, 2015; Altay et al., 2016). However, none of these prior studies showed *Glycyrrhiza* to be monophyletic, and all suffered from under-sampling (i.e., a maximum of seven species) and, therefore, did not represent strong molecular evidence. Thus, the infra-generic phylogeny of *Glycyrrhiza* and its relationship to *Glycyrrhizopsis* and *Meristotropis* require further investigation.

*Glycyrrhiza* has a widespread distribution, in every continent except for Antarctica (Kruganova, 1955; Lock and Schrire, 2005; Meng, 2005 also see the map in Figure 3), but it is widely distributed in north temperate zones globally, with only sparse distribution in the south temperate zone. On the other hand, most of the species of *Glycyrrhiza* are in the Old World, covering a vast area from the western Mediterranean region (Iberian peninsula in Europe and Algeria in North Africa), through the Russian Far East, Mongolia and northern China (as well as Sichuan and Yunnan of southwestern China) (Yeo, 1968; Chamberlain, 1970; Townsend, 1974; Ali, 1977; Rechinger, 1984; Lock, 1989; Podlech, 1991; Yakovlev, 1996; García, 1999; Bao and Larsen, 2010) to southern Australia (only *G. acanthocarpa* J.M.Black), including the states of Queensland, South New Wales, Victoria, South Australia, and Western Australia (Stanley and Ross, 1983; Weber, 1986; Gardner, 1991; Jeanes, 1996; Western Australian Herbarium, 1998). Only two species naturally occur in the New World: *Glycyrrhiza lepidota* in western temperate Canada and the United States (Britton and Brown, 1897; Scoggan, 1978) and *G. astragalina* Gillies in the temperate region of Argentina and Chile (ca. 40°S; Reiche, 1898; Gómer-Sosa, 1999). In contrast, the genera *Glycyrrhizopsis* and *Meristotropis* are restricted in southern Anatolia of Turkey (Chamberlain, 1970; Çetin, 2015) and Central Asia (Kruganova, 1955; Meng, 2005), respectively.

Notably, there are three classic types of intercontinental disjunction patterns represented by the distributional area of *Glycyrrhiza*: Eurasian-North American disjunction (i.e., north temperate disjunction, sensu Raven, 1972) North-South American amphitropical disjunction (trans-tropical disjunction, sensu Raven, 1972) and Asian-Australian disjunction (Southern Hemisphere temperate disjunction, sensu Kruganova, 1955; Raven, 1972; Meng, 2005). Compared to the other two types, the pattern of Asian-Australian disjunction is relatively unusual among angiosperm genera (for some examples see Gussarova et al., 2008; Nie et al., 2012; Temirbayeva and Zhang, 2015; Kramina et al., 2016; Slovák et al., 2018). On the other hand, many intercontinental diusjunctions comprise trans-tropical disjunctions sensu Raven (1972) or north and south temperate pattern sensu Thorne (1972). Wu et al. (2003, 2006) speculated that *Glycyrrhiza* may have originated in the Tethys region, and dispersed to eastern Gondwana, suggesting a fairly ancient series of events. In contrast, more recent inferences in other lineages (Apodanthaceae see Bellot and Renner, 2014; *Nitraria* see Temirbayeva and Zhang, 2015) based on molecular dating have shown much younger ages for similar patterns of disjunction (e.g., as predicted by Raven, 1972) compared to those presumed by Wu et al. (2006). Thus, the historical biogeography and the causes of the intercontinental disjunction of *Glycyrrhiza* needs further investigation using current analytical tools, and the subsequent findings may yield a greater understanding of evolutionary and biogeographic processes in other, similarly, widely distributed plant taxa.

In the present study, we inferred the phylogeny of *Glycyrrhiza* s.l. using dense taxonomic sampling and molecular sequence data from whole chloroplast (cp) genomes to (1) reveal the infra-generic relationships of *Glycyrrhiza* and test the phylogenetic positions of *Glycyrrhizopsis* and *Meristotropis* and (2) estimate the divergence times of lineages and reconstruct their biogeographic history. This study will provide a robust phylogenetic framework for resolving taxonomic problems in *Glycyrrhiza* as well as for sustainably using and developing consumable products from the genus.

## Materials and Methods

### Taxon Sampling

Sampling for molecular phylogenetic analyses consisted of 58 accessions representing 22 species of *Glycyrrhiza* s.l., including all the species, except for the rare *Glycyrrhizopsis syriaca* Turrill, accepted by Meng (2005) which is considered as the most thorough revision of *Glycyrrhiza* s.l. Outside of *Glycyrrhiza* s.l., within the IRLC clade, we sampled eight species representing five genera within tribe Wisterieae sensu Compton et al. (2019), and six species representing six other genera of the IRLC as in LPWG (2013). Four species representing three genera for the robinioids clade (sensu Wojciechowski et al., 2004) were sampled. We also included *Hylodesmum podocarpum* (Candolle) H.Ohashi & R.R.Mill as the outgroup. Most sequences for the study were obtained from field-collected or herbarium specimens and were new to this study (72 accessions, 38 species; see Supplementary Table S1 for detalis). DNA samples of *Austrocallerya megasperma* (F.Muell.) J.Compton & Schrire, *Glycyrrhiza astragalina* – 1 and *Wisteria floribunda* (Willd.) DC. were obtained from the DNA and Tissue Bank, Kew^1^. In addition, we downloaded sequences of *Lotus japonicus* (Regel) K.Larsen and *Robinia pseudoacacia* L. – 2 from GenBank (see Supplementary Table S1 for details).

### DNA Extraction, Genome Assembly, Annotation, and Alignment

We extracted the total genomic DNA following a modified CTAB protocol (Doyle and Doyle, 1987). Yield and integrity (size distribution) of genomic DNA extracts were quantified by fluorometric quantification on a Qubit (Invitrogen, Carlsbad, California, United States) using a dsDNA HS kit, as well as by visual assessment on 1% agarose gels. Subsequently, we used all samples to build blunt-end DNA libraries using the NEBNext Ultra II DNA library Prep kit for Illumina (New England Bio-labs) following the protocol of the manufacturer. We pooled the final indexed libraries in equimolar ratios and sequenced them in a single lane of an Illumina XTen sequencing system (Illumina Inc.).

From the raw reads, we filtered out adaptors and low-quality reads in Trimmomatic v.0.33 (Bolger et al., 2014). We checked the quality of the remaining reads using FastQC^2^ and performed *de novo* assembly in SPAdes 3.11 (Bankevich et al., 2012) with the k-mer of 75, 85, 95, and 105. We employed a customized python script (Jin et al., 2018) with its default parameters to apply BLAST and a built-in library to connect verified contigs into plastomes in SPAdes. We annotated the assembly of the resulting complete chloroplast (cp) genomes using the Dual Organellar GenoMe Annotator (DOGMA) (Wyman et al., 2004) with *Glycyrrhiza glabra* (GenBank Accession #: NC_024038; Sabir et al., 2014) as a reference. Most of our samples belonged to the IRLC (Wojciechowski et al., 2004) which lack the inverted repeat (IR) regions (Lavin et al., 1990). Thus, to better align with the cp genomes of *Glycyrrhiza* s.l., IR regions of *Hylodesmum podocarpum*, *Lotus japonicus*, *Robinia pseudoacacia*, and *Sesbania cannabina* (Retz.) Poir. were removed. We also extracted 75 protein coding sequences (CDSs; as in Kang et al., 2018 except for *rps12*) from the annotated genome and concatenated them with Geneious Prime (Kearse et al., 2012).

### Phylogenetic Analyses

We aligned whole cp genomes and CDSs independently with MAFFT v.7 (Katoh and Standley, 2013). We partitioned the alignment of CDSs but regarded the whole cp genomes without partitioning because intergenic spacers cannot be reliably modeled independently and based on many other recent studies (Wei et al., 2017; Wen et al., 2018; Yang et al., 2019). The best nucleotide substitution models of the whole cp genome and each CDS were detected using PartitionFinder 2 (Lanfear et al., 2016) under the default settings. GTR+G was selected as the best model for the whole cp genome and the best model for each CDS are shown in Supplementary Table S2.

We carried out separate phylogenetic analyses based on the whole cp genomes and the combined CDSs using Bayesian inference (BI; Rannala and Yang, 1996; Mau et al., 1999) implemented in the program MrBayes 3.2.5 (Ronquist and Huelsenbeck, 2003; Ronquist et al., 2012) by applying default prior settings. Each BI was performed using two independent runs of the Markov chain Monte Carlo (MCMC) for 10 million generations with sampling every 1,000 generations. We discarded the first 2,500 trees as burn-in and summarized the remaining posterior topologies as a maximum clade credibility (MCC) tree. We verified stationarity of the analyses with Tracer v1.6^3^ by ensuring that all ESS values exceeded 200 and we confirmed convergence between independent runs. In addition to BI, we also performed maximum likelihood (ML) analyses using IQ-TREE v.1.6 (Nguyen et al., 2015) with the following settings: rapid bootstrap analysis with 1,000 replicates followed by a search for best-scoring ML tree starting with a random seed. We observed that both the BI and ML trees generated from the whole cp genomes had identical topologies but higher support values than those from CDSs (see Supplementary Trees 1, 2; see additional reasons in Caption and Notes in Supplementary File). Therefore, we used the whole cp genomes in subsequent phylogenetic and biogeographic analyses.

### Divergence Time Estimation

We performed divergence time dating with a lognormal relaxed clock model in BEAST v.2.6.1 (Bouckaert et al., 2019) based on an alignment with no more than three accessions per species within *Glycyrrhiza* s.l. (see Alignment 2 and Caption and Notes in the Supplementary File). We used default parameters except that we applied the birth-death model for tree branching processes, the nucleotide substitution model of GTR+G based on PartitionFinder 2, and constrained the ages of two nodes. We constrained the root age at 50.6 Ma based on the results of Lavin et al. (2005). In addition, and set the stem age of *Wisteria* Nutt. according to the oldest reliable fossil with both fruits and leaves described by Wang et al. (2006). The fossil, *Wisteria shanwangensis* Wang, Dilcher, Zhu, Zhou et Lott, occurs in the Middle Miocene Shanwang flora, China, which has been recently dated to ca. 17 Ma on the basis of ^40^Ar/^39^Ar analysis (He et al., 2011). The prior distributions of the both calibration points were set as log normal, with a standard deviation of 1.0. Throughout, we follow the geological time scale according to the chronostratigraphic chart of International Commission on Stratigraphy (ICS)^4^ for the names of geological units and their time durations.

The parameters of BEAST were set using BEAUti v.2.5.0 (Bouckaert et al., 2019) with the abovementioned fossil calibrated nodes constrained as monophyletic according to Wojciechowski (2003) and Wojciechowski et al. (2004), and our present phylogenetic results. In BEAST, we performed the MCMC run for 700 million generations with sampling every 1,000 generations. Checking with the program of Tracer, the analysis reached convergence with burn-in of the first 170 million states. We performed the burn-in and then determined the MCC tree with mean heights in TreeAnnotator. The MCC tree was annotated as a chronogram with mean ages for the nodes and 95% highest posterior density (HPD) intervals.

Before conducting the analysis in BEAST, we determined the effective priors by performing an analysis with priors only (i.e., without sequence data). The analysis with priors only is important for revealing the actual marginal distributions of priors, which may differ from those set for the analysis due to complex interactions with other calibrations and the tree process, i.e., birth-death model, herein (Heled and Drummond, 2012; Warnock et al., 2015; Muellner-Riehl et al., 2016). In the case of the calibration of the two crown nodes, the marginal distribution of the priors reflected our intended settings, indicating limited prior interactions (see Prior-only.log in the Supplementary Material).

### Ancestral Area Reconstruction

To infer the ancestral areas of *Glycyrrhiza* s.l., we used the Dispersal-Extinction-Cladogenesis model (DEC; Ree and Smith, 2008) implemented within a statistical framework in RASP v.4.0 (Yu et al., 2015). According to the distribution records of *Glycyrrhiza* s.l. in Kruganova (1955) and Meng (2005), and Li and Lu (2015) and taking endemism into consideration (Linder, 2001), we coded the extant taxa so that species fell into one or two geographic areas (see the map in Figure 3): (A) eastern Asia: northern China (except for Inner Mongolia and Xinjiang), southwestern China (Sichuan and Yunnan) and the Russian Far East; (B) interior of Eurasia (east of Black Sea): southern Siberia, Mongolia, Inner Mongolia and Xinjiang of China, Central Asia, western Himalayan region, northern Iran, western Asia (except for Turkey and Mediterranean east coast), Caucasus and European Russia; (C) circum-Mediterranean region: southern Europe, Turkey, Mediterranean east coast and northern Africa; (D) Australia: southern Queensland, South New Wales, Victoria, eastern South Australia and southeastern Western Australia; (E) temperate western North America: regions of west of the Great Lakes in United States and Canada; (F) temperate South America: around 40°S in Argentina and Chile. To infer the ancestral areas, we obtained 300,000 post-burnin trees and the corresponding MCC tree from the abovementioned BEAST analysis. One accession per species had remained within *Glycyrrhiza* s.l. (details see Caption and Notes in Supplementary File) and all the other taxa were pruned with Ape library in R (Paradis and Schliep, 2018) as including sparsely sampled related lineages is ill-advised and can lead to erroneous inferences at the root (Ronquist, 1997; Harris et al., 2013). All parameters were set as default in RASP except that the maximum number of areas was constrained to two based on the maximum number of areas in which extant species occur.

## Results

All 72 newly sequenced plastomes were successfully assembled into complete circular configurations. The sizes of the plastid genomes ranged from 122,421 to 156,702 bp and the GC contents were between 33.9 and 36.0%, respectively (see Supplementary Table S1 for details). The lengths of alignments for the analyses in MrBayes, IQ-TREE, and BEAST (see Alignment 1 and 2 in the Supplementary File) were 241,311 and 167,779 bp, respectively. The structure and gene order of *Glycyrrhiza* s.l. were identical as those reported in previous studies on several cp genomes of *Glycyrrhiza* (Raveendar et al., 2017; Kang et al., 2018).

### Phylogenetic Relationships

**FIGURE 1:**
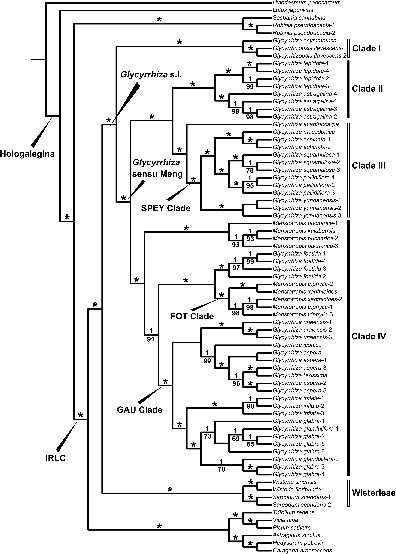
Maximum clade credibility tree resulting from Bayesian Inference of *Glycyrrhiza* s.l. based on whole chloroplast genome sequences. Bayesian posterior probabilities (*PP* ≥ 0.95) are given above branches, maximum likelihood bootstrap values (LBS ≥ 50%) below branches. Asterisks indicate both of *PP* = 1 and LBS = 100%. The Hologalegina and IRLC clades were labeled following Wojciechowski et al. (2004).

We present the phylogenetic tree with Bayesian posterior probabilities (PP) and ML bootstrap (LBS) values on the BI tree branches (Figure 1; BI tree with branch lengths see Supplementary Tree 3; ML tree see Supplementary Tree 4). The phylogenetic result indicated that *Glycyrrhiza* s.l. was monophyletic (*PP* = 1, LBS = 100%), forming a clade (*PP* = 1, LBS = 100%) with Wisterieae (*PP* = 1, LBS = 100%). This large clade was sister to all other sampled IRLC.

We recovered four main clades within *Glycyrrhiza* s.l. (clades I–IV), all of which were highly supported with *PP* = 1, LBS = 100%. Clade I contained *Glycyrrhizopsis flavescens* (*PP* = 1, LBS = 100%) and *Glycyrrhiza asymmetrica* Hub.-Mor., and was sister to all other *Glycyrrhiza* s.l., which correspond to *Glycyrrhiza* sensu Meng (Figure 1; *PP* = 1, LBS = 100%). Within *Glycyrrhiza* sensu Meng, Clade II was comprised of two well supported species, *Glycyrrhiza astragalina* (*PP* = 1, LBS = 100%) and *G. lepidota* (*PP* = 1, LBS = 100%). Within Clade III, *G. acanthocarpa* was sister to all other species, comprising *G. echinata*, *G. macedonica* Boiss. & Orph., *G. pallidiflora* Maxim., *G. squamulosa* Franch., and *G. yunnanensis* Cheng f. & L.K.Tai ex P.C.Li, which formed a clade (*PP* = 1, LBS = 100%) and was regarded as the “SPEY clade” hereafter. Clade IV consisted of 13 species and included a nested clade comprising *Meristotropis bucharica* (Regel) Kruganova and *M. kulabensis* Masl. (*PP* = 1, LBS = 100%), which were sister to the rest of Clade IV (*PP* = 1, LBS = 91%). The next diverging clade within Clade IV comprises *G. foetida* Desf., *Meristotropis triphylla* Fisch. & C.A.Mey, and *M. xanthioides* Vassilcz (*PP* = 1, LBS = 100%; as the “FOT clade”) and the remaining eight species constituted the “GAU clade” (*PP* = 1, LBS = 100%).

### Divergence Times

**FIGURE 2:**
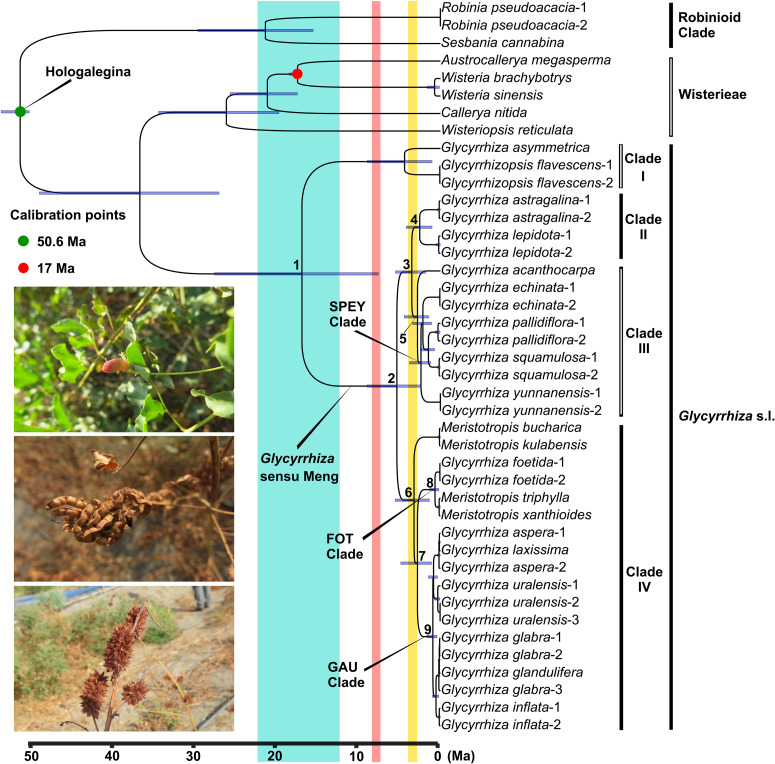
Ultrametric chronogram from BEAST of *Glycyrrhiza* s.l. and related groups based on chloroplast genome data. Blue bars represent the 95% highest posterior density credibility interval for node ages. Three paleogeological events are indicated with colored vertical rectangles: uplift of the Turkish-Iranian plateau (blue), the third rapid elevation of Qinghai-Tibetan Plateau (QTP; pink), and the fourth rapid upraising of QTP (yellow). The three photos show the fruits of *Glycyrrhiza inflata* **(upper)**, *G. uralensis* **(middle)**, and *G. pallidiflora* **(lower)**, respectively.

All the PP values on our BEAST tree are 1.0, except for the *Glycyrrhiza* s.l.-Wisterieae clade (*PP* = 0.91; see Supplementary Tree 5). According to our molecular dating result (Figure 2), the ancestor of *Glycyrrhiza* s.l. originated at 37.02 Ma (95% HPD: 27.26–49.39 Ma) during the late Eocene, and Clade I and *Glycyrrhiza* sensu Meng diverged at 17.05 Ma (Node 1, 95% HPD: 7.64–27.83 Ma). The split of the ancestor of Clades II and III and that of Clade IV was dated to 5.36 Ma (Node 2, 95% HPD: 2.44–9.03 Ma) around the Miocene-Pliocene boundary, followed by the divergence of Clade II and Clade III at 3.53 Ma (Node 3, 95% HPD: 1.81–5.54 Ma). Within Clade II, the South American *Glycyrrhiza astragalina* and North American *G. lepidota* split at 2.55 Ma (Node 4, 95% HPD: 1.03–4.23 Ma). On the other hand, at around 2.82 Ma (Node 5, 95% HPD: 1.45–4.46 Ma), the most recent common ancestors (MRCA) of the Australian *G. acanthocarpa* separated from the SPEY clade within Clade III. The crown group of Clade IV began to diverge in the late Pliocene (Node 6, 3.26 Ma, 95% HPD: 1.36–5.58 Ma). Subsequently, the FOT clade and the GAU clade diverged at 2.83 Ma (Node 7, 95% HPD: 1.11–4.88 Ma). More recently, the FOT clade and the GAU clade started to split at 0.67 Ma (Node 8, 95% HPD: 0.19–1.34 Ma) and 0.93 Ma (Node 9, 95% HPD: 0.42–1.61 Ma), respectively.

### Ancestral Area

**FIGURE 3:**
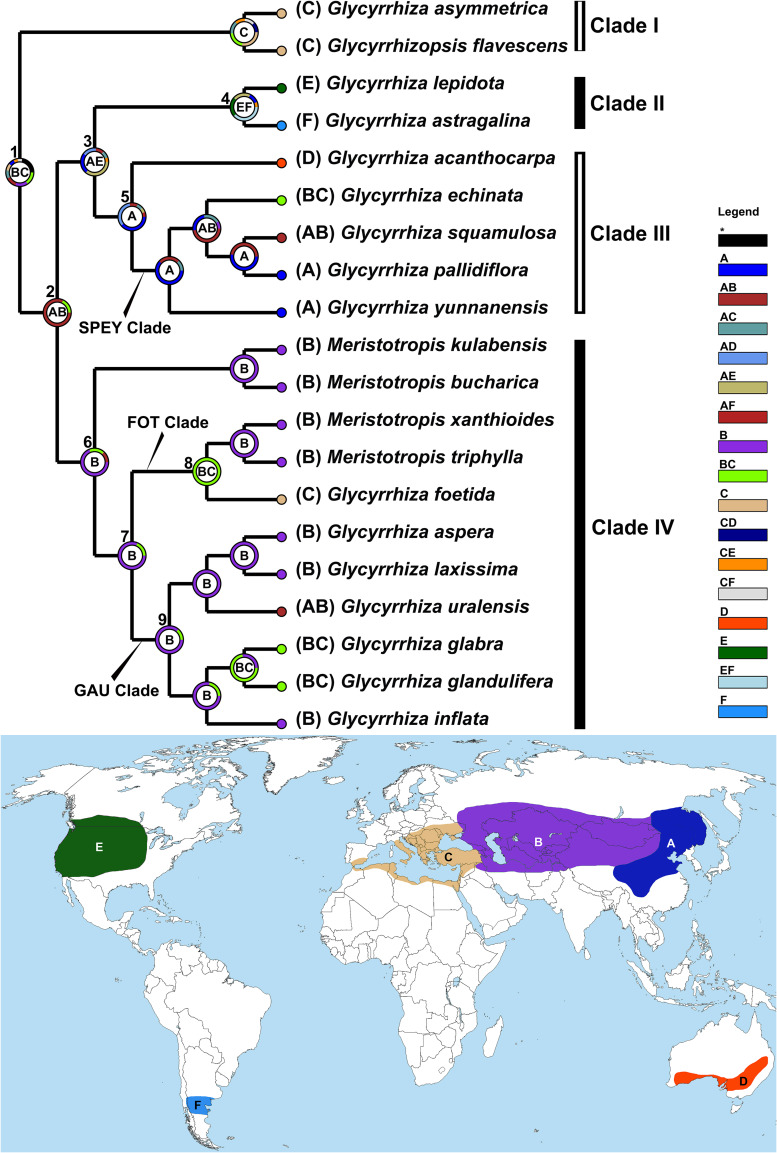
Ancestral area reconstruction of *Glycyrrhiza* s.l. estimated under the DEC model implemented in RASP v.4.0 based on the tree obtained from BEAST. The most likely ancestral distributions are labeled in the circle of nodes. The map shows the coding areas in different colors. For each species with multiple accessions, the “# 1” accessions were selected for this analysis (details see Supplementary Table S1).

Our DEC analysis (Figure 3; also see DEC result in the Supplementary File) indicated the interior of Eurasia and the circum-Mediterranean regions as the ancestral range of *Glycyrrhiza* s.l. (Node 1; coding: BC). The MRCA of was Clade I was restricted in circum-Mediterranean region (coding: C), and that of *Glycyrrhiza* sensu Meng spanned from Russian Far East and northern China to the Caucasus region (Node 2; coding: AB). *Glycyrrhiza* sensu Meng split into the MRCA of Clades II and III, spanning eastern Asia and North America (Node 3; coding: AE), and that of Clade IV (Node 6), which was distributed in the interior of Eurasia (coding: B). Subsequently, the MRCA of Clade II expanded to South America (Node 4; coding: EF), while the ancestor of Clade III remained in eastern Asia (Node 5; coding: A) and subsequently migrated to Australia (*Glycyrrhiza acanthocarpa*; coding: D). As for Clade IV, most of taxa were confined in the interior of Eurasia (coding: B), while a few groups, including the MRCA of the FOT clades (Node 8), and the MRCA of *G. glabra* and *G. glandulifera* Waldst. & Kit., spread back to the circum-Mediterranean region (coding: BC). Also within Clade IV, *G. uralensis*, dispersed to eastern Asia (coding: AB).

## Discussion

### Phylogenetic Relationships of *Glycyrrhiza* s.l.

As shown in previous studies, *Glycyrrhiza* belongs to the IRLC (inverted repeat lacking clade; Lavin et al., 1990; Wojciechowski et al., 2000, 2004; Lock and Schrire, 2005) and more broadly to the Hologalegina, a larger group including IRLC and the robinioid clade (Wojciechowski, 2003; Lavin et al., 2005; Farruggia and Howard, 2011; Cardoso et al., 2012, 2013; also see Figure 1). Our analyses suggested that a large clade comprising *Glycyrrhiza* s.l. and Wisterieae sensu Compton et al. (2019) was sister to the rest of the IRLC (Figure 1). In previous taxonomic studies, *Glycyrrhiza* was treated within tribe Galegeae (Grigorev and Vasilchenko, 1948; Chamberlain, 1970; Bao and Larsen, 2010), however, our results show that Galegeae is non-monophyletic, with *Glycyrrhiza* and *Astragalus strictus* R.Grah. ex Benth distantly related, consistent with recent molecular phylogenetic studies (Wojciechowski et al., 2004; LPWG, 2013; Duan et al., 2015, 2016).

Within *Glycyrrhiza* s.l., we recognized four main clades (Figure 1) that are consistent with earlier studies having more limited sampling of the genus (Erayman et al., 2014; Çetin, 2015; Altay et al., 2016). Clade I includes *Glycyrrhizopsis flavescens* and *Glycyrrhiza asymmetrica* (Figure 1) and is sister to *Glycyrrhiza* sensu Meng. Therefore, it may be appropriate to treat *G. asymmetrica* in *Glycyrrhizopsis*. However, as our present study is based on a single accession of *G. asymmetrica*, it is unclear whether the species should be merged with *Glycyrrhizopsis flavescens*.

The monophyletic *Glycyrrhiza* sensu Meng contains Clades II, III, and IV and is sister to Clade I. The species of Clade II and III are distinguished on the basis of thorny, globular to ellipsoid fruits (except for *G. squamulosa* with prickless pods; see Grigorev and Vasilchenko, 1948; Jeanes, 1996; Li and Cui, 1998). Clade II includes *Glycyrrhiza astragalina* and *G. lepidota* (Figure 1), which are restricted in South and North America, respectively (Meng, 2005). This clade was resolved as sister to Clade III, and that result is new to this study.

The core taxa of Clade III are distributed in temperate Eurasia (Kruganova, 1955; Li and Lu, 2015), however, *G. acanthocarpa* ranges to southern Australia and diverged first in this clade (Figure 1). Within Clade III, *G. pallidiflora* is not sister to *G. yunnanensis*, which supports treating these as separate species (Grankina, 2008; Bao and Larsen, 2010; Li and Lu, 2015) rather than one species as was proposed by Meng (2005). On the other hand, *Glycyrrhiza macedonica*, represented by a single accession, and *G. echinate*, represented by two accessions, were sisters so that the controversial species status of the former (Grigorev and Vasilchenko, 1948; Kruganova, 1955; Li, 1963; Meng, 2005; Li and Lu, 2015; Çetin, 2015) cannot be determined by our study (Figure 1). *Glycyrrhiza macedonica* was circumscribed within *G. echinata* by Meng (2005) and Çetin (2015) while Grigorev and Vasilchenko (1948), Kruganova (1955), Li (1963), and Li and Lu (2015) kept it as a separate species. Their species boundary needs to be further delimited in the future study with additional evidence.

Clade IV was previously recovered by Hayashi et al. (2005) and Meng (2005). This clade, exhibits diverse morphology, is the largest clade in *Glycyrrhiza* s.l., and is widely distributed in northern temperate regions of the Old World (Kruganova, 1955; Li and Lu, 2015). Our result (Figure 1) explicitly resolved three monophyletic species: *G. foetida*, *G. inflata* and *G. uralensis*. As for other species, we discover that *G. glandulifera* was embedded within the accessions of *G. glabra* and *G. iconica* Hub.-Mor. was sister to a complex with *G. laxissima* Vassilcz. that was within *G. aspera* Pall. Additionally, *Meristotropis kulabensis* was embedded within *M. bucharica*, and *M. xanthioides* was nested within *M. triphylla*. In these respects, our results are congruent with Meng (2005) and agree with circumscribing *G. glandulifera*, *G. laxissima*, *Meristotropis kulabensis*, and *M. xanthioides* as synonyms of *G. glabra*, *G. aspera*, *M. bucharica*, and *M. triphylla*, respectively. Moreover, it is notable that the “three-leaflets group,” formerly recognized as the genus *Meristotropis* by Vasilchenko (1948) and Kruganova (1955), is non-monophyletic according to our cp genome tree (Figure 1) and can be merged into *Glycyrrhiza* as suggested by Li (1963), Meng (2005), and Li and Lu (2015). The formal generic and species delimitation will be included in our taxonomy study in the near future.

### Historical Biogeography of *Glycyrrhiza* s.l.

According to our dating result (Figure 2) and ancestral area inferences (Figure 3), the ancestor of *Glycyrrhiza* s.l. originated during the late Eocene and its MRCA was likely distributed within the interior of Eurasia and circum-Mediterranean region. From the late Eocene to early Oligocene, the global temperature saw a dramatic decrease (Prothero, 1994; Zachos et al., 2001; Hansen et al., 2013) and the climate turned drier in Eurasia (Axelrod, 1975; Utescher and Mosbrugger, 2007; Sun et al., 2014), where the boreotropical flora, which had flourished in the early Cenozoic, was gradually replaced by sclerophyllous vegetation (Tiffney, 1985a, b; Mai, 1989). The climatic and vegetational change may lead to the separation between the MRCA of mesic/xeric, herbaceous *Glycyrrhiza* s.l. and Wisterieae, which are primarily thermophilic, woody lianas (Compton et al., 2019).

Our ancestral area reconstruction indicated that Clade I split from *Glycyrrhiza* sensu Meng in the Middle Miocene (Figure 2). The split was likely caused by a vicariance event that separated the MRCA of Clade I in the Mediterranean region and an ancestor of *Glycyrrhiza* sensu Meng in the interior of Eurasia. Thereafter, *Glycyrrhiza* sensu Meng was dispersed into eastern Asia before the clade diversified. In contrast, Clade I remained isolated in the Mediterranean region where extant species are endemic to the southern Anatolian plateau (Meng, 2005; Öztürk et al., 2017). The vicariance event leading to the separation of Clade I from *Glycyrrhiza* sensu Meng may have been the uplift of Turkish-Iranian Plateau (∼22–12 Ma; Mouthereau, 2011; Djamali et al., 2012) while the short period of warm climate in Middle Miocene (Middle Miocene Climatic Optimum; Böhme, 2003; You et al., 2009) may have facilitated the spread of *Glycyrrhiza* sensu Meng into eastern Asia (Figure 3).

Historical biogeography may help to elucidate the taxonomy of *Glycyrrhizopsis*. Most taxonomists treated *Glycyrrhizopsis* (corresponding to Clade I herein) within *Glycyrrhiza* (Li, 1963; Chamberlain, 1970; Li and Lu, 2015; Çetin, 2015), however, a few revisions raised *Glycyrrhizopsis* (Boissier, 1856; Meng, 2005) to generic status given the characters of yellow corolla, separated wings and dehiscent legumes. The geographic segregation of *Glycyrrhizopsis* from *Glycyrrhiza* in the Middle Miocene may have lent more support to the generic status of *Glycyrrhizopsis* as the two genera have seemingly developed in isolation for ca. 17 million years (Figure 2, Node 1).

The third rapid uplift of the Qinghai-Tibetan Plateau (QTP) occurred at 8–7 Ma, leading to a cooler climate and aridification of inland Asia (Harrison et al., 1992; Liu et al., 2001; Zheng and Yao, 2006; Wang et al., 2008) and subsequent psychrophytic and xerophytic adaptation of some temperate forest species, some of which developed shrub or herbaceous habits (Sun, 2002; Meng et al., 2015). Within *Glycyrrhiza* sensu Meng, two groups may have diverged within this paleoclimatic context around the Miocene-Pliocene boundary (Figure 2): the MRCA of Clades II and III, which was distributed in eastern Asia and North America, and the MRCA of Clade IV, which occurred in the interior of Eurasia (Figure 3).

*Glycyrrhiza* sensu Meng underwent rapid diversification and reached the New World and Australia within a short period during Pliocene. The ancestor of each of Clades II and III separated at ca. 3.53 Ma, and the crown groups of Clades II and III diverged at ca. 2.55 Ma and ca. 2.82 Ma, respectively (Figure 2). On the other hand, Clade IV, which contains more than half of the species of *Glycyrrhiza* sensu Meng, began divergence ca. 3.26 Ma (Figure 2). The initiation of the rapid diversification of *Glycyrrhiza* sensu Meng is possibly a response to the last (fourth) rapid uplift of the QTP. The uplift yielded aridification of the Asian interior and the formation of the Loess Plateau, which may have facilitated speciation of the Eurasian *Glycyrrhiza* sensu Meng (3.6–2.5 Ma; Chen et al., 1999; Li and Fang, 1999; Li et al., 2001; Tang and Liu, 2001; Zheng and Yao, 2006). The phenomenon of recent rapid diversification driven by climatic and habitat change is well-known by phytogeographers, and other examples can be seen within IRLC groups (*Gueldenstaedtia* and *Tibetia* in Xie et al., 2016; *Oxytropis* in Shahi Shavvon et al., 2017; *Astragalus* in Azani et al., 2019; *Hedysarum* in Nafisi et al., 2019) in papilionoid genera (*Ammopiptanthus* in Shi et al., 2017; *Sesbania* in Farruggia et al., 2018) and in angiosperm taxa (*Allium* and mint tribe Elsholtzieae in Li et al., 2016, 2017), respectively; *Globularia* and *Campylanthus* in Affenzeller et al. (2018), *Coptis* in Xiang et al. (2018), and *Paris* in Ji et al. (2019).

During the early Pliocene, species of Clade II migrated to temperate western North America (*Glycyrrhiza lepidota*; ca. 5.36–3.53 Ma; Figures 2, 3: Nodes 2–3), then arrived at temperate South America in the late Pliocene (*G. astragalina*; ca. 3.53–2.55 Ma; Figures 2, 3: Nodes 3–4) (Reiche, 1898; Gómer-Sosa, 1999; Meng, 2005). The ancestor of the Clade II was less likely to disperse directly from eastern Asia to South America (see map in Figure 3; but see Du et al., 2020), we suspect a migration route from eastern Asia to North America via the Bering land bridge (BLB). The timing of the migration may have coincided with BLB II (20–3 Ma), which is regarded as the main phase for trans-Beringian floristic exchanges (Wen et al., 2016; Duan et al., 2020). Some other papilionoid IRLC genera, such as *Astragalus* (Azani et al., 2019) and *Hedysarum* (Liu, 2017) were also found to have migrated through BLB from the early Pliocene to early Pleistocene. The disjunct pattern of *Glycyrrhiza lepidota* and *G. astragalina* comprises a temperate amphitropical disjunction (Raven, 1963; Wen and Ickert-Bond, 2009; Simpson et al., 2017) which may often result from bird-mediated long-distance dispersal (Popp et al., 2011). The rapid dispersal of *G*. *astragalina* from North America to South America (within one million years, see above) seems to favor an LDD hypothesis rather than one of gradual range expansion. This is especially true due to intervening habitats across the Americas that may have been unsuitable for the ancestor of this group and would have served a barrier to gene flow along with distance following an LDD, also supporting the rapid speciation event (Harris et al., 2018).

Within Clade III, the MRCA of *Glycyrrhiza acanthocarpa* and the SPEY clade diverged within the late Pliocene (Figure 2), and, subsequently, *G. acanthocarpa* reached southern Australia (Kruganova, 1955; Meng, 2005; see Figure 3). The pattern of Asian-Australian disjunction is relatively uncommon within angiosperms. Thorne (1972) who regarded the pattern as “North and South Temperate disjunction,” speculated its possible causes including “extinction of montane species formerly spanning the gaps” or due to bird migration. Although we cannot completely rule out the scenario that *Glycyrrhiza* arrived at Australia by a stepwise colonization directed from Southeast Asia via Indo-China region and Indonesia archipelago (Raven and Axelrod, 1974), the vast area of the tropical and subtropical regions between northern eastern China and southern Australia, which is uninhabitable to *Glycyrrhiza*, significantly decreases this potential (Heaney, 1991; Kershaw, 1994) over the relatively short time frame involved in the evolution of this genus. Thus, we are inclined to think that the colonization of Australia from an eastern Asian ancestor may result from a single or a few bird-mediated LDD events (Holzapfel, 1994; see Figure 3: A, Node 5). Notably, more Australian plant genera may have originated from other continents, i.e., from South America (Nie et al., 2012) and Africa-western Asia (Temirbayeva and Zhang, 2015; Kramina et al., 2016) compared to eastern Asia. Therefore, our study provides a case of this uncommon pattern of the Asia-originated Asian-Australian disjunct distribution, such as reported in Slovák et al. (2018) for *Picris* (Compositae). A more common pattern was observed for SPEY clade, for which the MRCA was located in eastern Asia (Figure 3) with subsequent spread back to the interior of Eurasia and the Mediterranean region (Figure 3) during the early Pleistocene (since 2.4 Ma; Figure 2).

Similar to the case of separation of Clades II and III (see Discussion above and Figures 2, 3), Central Asian aridification induced by the fourth rapid elevation of QTP may also have led to the split within Clade IV (Figure 2). The MRCA of Clade IV occurred in the interior of Eurasia and, while its two descended lineages, *Meristotropis bucharica* and the ancestor of the rest of Clade IV remained in the same area (Figure 3). As the second diverging group within Clade IV, the MRCA of the FOT clade migrated from the interior of Eurasia to the Mediterranean region (Figure 3). A vicariance event then occurred within the FOT clade during the Middle Pleistocene (ca. 0.67 Ma; Figure 2), so that *Meristotropis triphylla* and *Glycyrrhiza foetida* were confined in Central Asia and North Africa, respectively (Kruganova, 1955; Meng, 2005; Li and Lu, 2015). The emergence of the Central Asian glacier in Pleistocene (Abramowski et al., 2006) may have served as a climatic barrier between these two species leading to their speciation in allopatry.

Within the GAU clade, we noticed that *Glycyrrhiza uralensis* is sister to a clade formed by *G. aspera* and *G, iconica* with insignificant support (Figure 1). Species of the GAU clade have more or less overlapping distributions (Kruganova, 1955; Meng, 2005) and previous studies reported that natural hybridization frequently occurred among these taxa (Ashurmetov, 1996; Chen et al., 2017; Kang et al., 2018), which may cause the weakly supported topology. Thus, it might be premature to structure the whole biogeographic story for the GAU clade at this stage, but we can still uncover its dispersal directions from interior of Eurasia (Figure 3) eastwards to eastern Asia (*G. uralensis*) and westwards to Mediterranean region (*G. glabra* and *G. glandulifera*) within the last million years (since 0.93 Ma; Figure 2). Nearly all of the medicinal liquorice species belong to the GAU clade (Chinese Pharmacopoeia Commission, 2015; Öztürk et al., 2017) but its accurate phylogenetic relationships and divergence/hybridization history need further studies based on a dense populational sampling and reliable nuclear sequences.

## Conclusion

The liquorice genus *Glycyrrhiza* has broad cultural and medicinal uses within Eurasia and the North America. This study presents the first comprehensive analysis of its evolutionary and biogeographic history using a phylogenomics approach with extensive sampling within *Glycyrrhiza* s.l. Our phylogenetic results support the monophyly of *Glycyrrhiza* s.l., inclusive of the satellite genera, *Glycyrrhizopsis* and *Meristotropis*. *Glycyrrhizopsis* appears phylogenetically distinct from *Glycyrrhiza* sensu Meng (2005) which includes *Meristotropis*. We suggest that *Glycyrrhiza* s.l. may have originated during the late Eocene, and its MRCA was distributed in the interior of Eurasia and the circum-Mediterranean region. Thereafter, the uplift of Turkish-Iranian and Qinghai-Tibetan Plateaus may be the main driver of rapid diversification of the major clades in *Glycyrrhiza* s.l. Since Pliocene, three liquorice species reached the temperate regions of North America, South America and Australia, respectively, via long-distance dispersal. Within *Glycyrrhiza* sensu Meng, the Asian-Australian disjunction is relatively rare in angiosperms, so that our study represents an important documentation of this pattern. Our results provide a robust theoretical foundation for sustainable utilization of genetic, pharmacological, and cultural resources of the economically significant liquorice genus and may be helpful elucidating evolutionary processes leading to broad intercontinental disjunctions in plants.

## Data Availability Statement

The datasets presented in this study can be found in online repositories. The names of the repository/repositories and accession number(s) can be found in the article/Supplementary Table S1.

## Author Contributions

LD and JW designed the research. H-FC financially supported this study. LD, EA, KE, PL, H-FC, and HH collected the samples. Z-RZ carried out the experiment. AH and CS performed the data analysis. LD, AH, and JW discussed the results and wrote the draft. All authors agreed on the contents of the final manuscript.

## Conflict of Interest

The authors declare that the research was conducted in the absence of any commercial or financial relationships that could be construed as a potential conflict of interest.

## Footnotes

1 https://dnabank.science.kew.org

2 www.bioinformatics.babraham.ac.uk/projects/fastqc/

3 http://tree.bio.ed.ac.uk/software/tracer

4 http://www.stratigraphy.org/
